# Initial clinical experience with a novel flexible endoscopic robot for transanal surgery

**DOI:** 10.1007/s10151-022-02577-1

**Published:** 2022-01-29

**Authors:** M. Morino, E. Forcignanò, A. Arezzo

**Affiliations:** Department of Surgical Sciences, University of Turin, C.so Dogliotti 14, 10126 Turin, Italy

**Keywords:** Colorectal cancer, Robotic surgery, Endoluminal surgery, Transanal endoscopic microsurgery

## Abstract

**Background:**

The Flex^®^ Robotic System (Medrobotics, Raynham, MA, USA) is the first miniaturised flexible endoscopic robot that aims to allow surgical manoeuvres beyond the area currently reached by transanal endoscopic microsurgery. The aim of this study is to evaluate our initial clinical experience with this novel tool.

**Methods:**

We prospectively collected all consecutive cases of local excisions of rectal lesions performed with the Flex^®^ Robotic System performed at the Department of Surgical Sciences of the University of Turin between October 2018 and December 2019. Indications were benign, or early rectal lesions judged unsuitable for endoscopic removal, within 20 cm of the anal verge. Debriefing meetings after each procedure allowed technology assessment leading to the modification, development, and implementation of tools according to the clinical experience. We analysed the data in terms of the safety and efficacy of treatment.

**Results:**

Between October 2018 and February 2020, 26 patients were treated. We performed a full-thickness excision in 14 patients and a submucosal dissection in 12. The median operating time was 115 min (range 45–360 min). In six patients (23.1%), we converted to standard transanal endoscopic operation (TEO^®^) (Karl Storz, Tuttlingen, Germany) to complete the procedure. The 30-day morbidity rate was 11.5% (3/26). Positive resection margins were detected in 4 (15.4%) patients. At a minimum follow-up of 12 months, 2 (7.7%) local recurrences were observed.

**Conclusions:**

This first clinical series demonstrates that the Flex^®^ Robotic System is a fascinating technology that deserves further development to increase surgical dexterity, thereby overcoming current technical limitations and improving clinical outcomes.

**Supplementary Information:**

The online version contains supplementary material available at 10.1007/s10151-022-02577-1.

## Introduction

According to the document "Against Cancer" on cancer screening in the European Union (2017) [1], colorectal cancer (CRC) is one of the three most frequent cancers in the world. With about 1.85 million new diagnoses in 2018, it is estimated that the incidence of CRC will double by 2030 [2–5]. Mass screening programs by colonoscopy have been established in most western countries. The introduction of less invasive screening tools will encourage further compliance with these programs. This will reduce the need for radical surgery favouring local excision and, therefore, endoluminal therapies.

Today only small, flat polyps (< 2 cm) can be resected en-bloc by snare using a conventional colonoscope. When attempts are made to resect larger tumours via endoscopic mucosal resection (EMR) there is a risk of dividing the tumour into fragments and spreading cancer cells. The impossibility of performing correct pathology examination and staging means that a second procedure may be needed in up to 20% of the cases, to ensure that the neoplasm has been completely removed as a precautionary measure [6, 7]. Endoscopic submucosal dissection (ESD) was developed 20 years ago in an attempt to resect lesions in one piece, reducing the risk of cancers spreading and permitting precise pathological staging. Nevertheless, ESD is technically demanding, and is not widely [8]. Transanal endoscopic microsurgery (TEM) is a rigid endoscopic platform designed 40 years ago, proving advantageous in most cases of local excisions of early rectal cancer [9]. However, TEM it can only be performed up to 15 cm into the rectum. More recently, transanal minimally invasive surgery (TAMIS) was introduced based on the use of a single-port introduced via the anus [10]. This has the merit of having made the transanal endoscopic technique of common use because it does not necessitate an investment of money into the TEM equipment1, without however a real benefit for the patient. [11, 12]. Despite the use of laparoscopy, colonic resection is associated with perioperative morbidity of up to 30% [13] and should be confined to indications unsuitable for endoscopic resections.

In this scenario, an appropriate technological step forward is needed. Therefore, Medrobotics (Medrobotics, Raynham, MA, USA) developed a novel, versatile robotic device, first for transoral and later for transanal application with the aim of improving treatment thanks to minimal invasiveness, and able to perform surgical procedures in narrow cavities and in locations that have been so far out of reach.

We evaluate the technology and the technique in our series of transanal local excisions performed with the Flex^®^ Robotic System.

## Materials and methods

The present study was a retrospective analysis of a prospective database created in October 2018. Indications for Flex^®^ Robot System were benign rectal lesions or early rectal cancer judged unsuitable for endoscopic removal. Lesions were considered suitable for Flex^®^ Robotic System treatment only when located within 20 cm of the anal verge at rigid proctoscopy, independently from the location on the circumference and the consequent risk of opening the peritoneum.

The procedures were all performed with original Flex^®^ Robotic System equipment in the presence of engineers of the Medrobotics company as in a learning curve. A briefing and a debriefing meeting were conducted before and at the end of each procedure. Technology assessment led to the modification, development, and implementation of tools according to the clinical experience of the surgeons. All devices were Conformité Européenne (European conformity [CE]) approved before introduction into the clinical practice. Before using, the Flex® Robotic System on patients the authors underwent 2-day specific training first on models, then ex vivo, and finally, human cadaver models for training in part of the procedure such as suturing, until they were able to complete a full procedure including dissection and suturing.

### *The Flex*^®^* Robotic System*

**Fig. 1:**
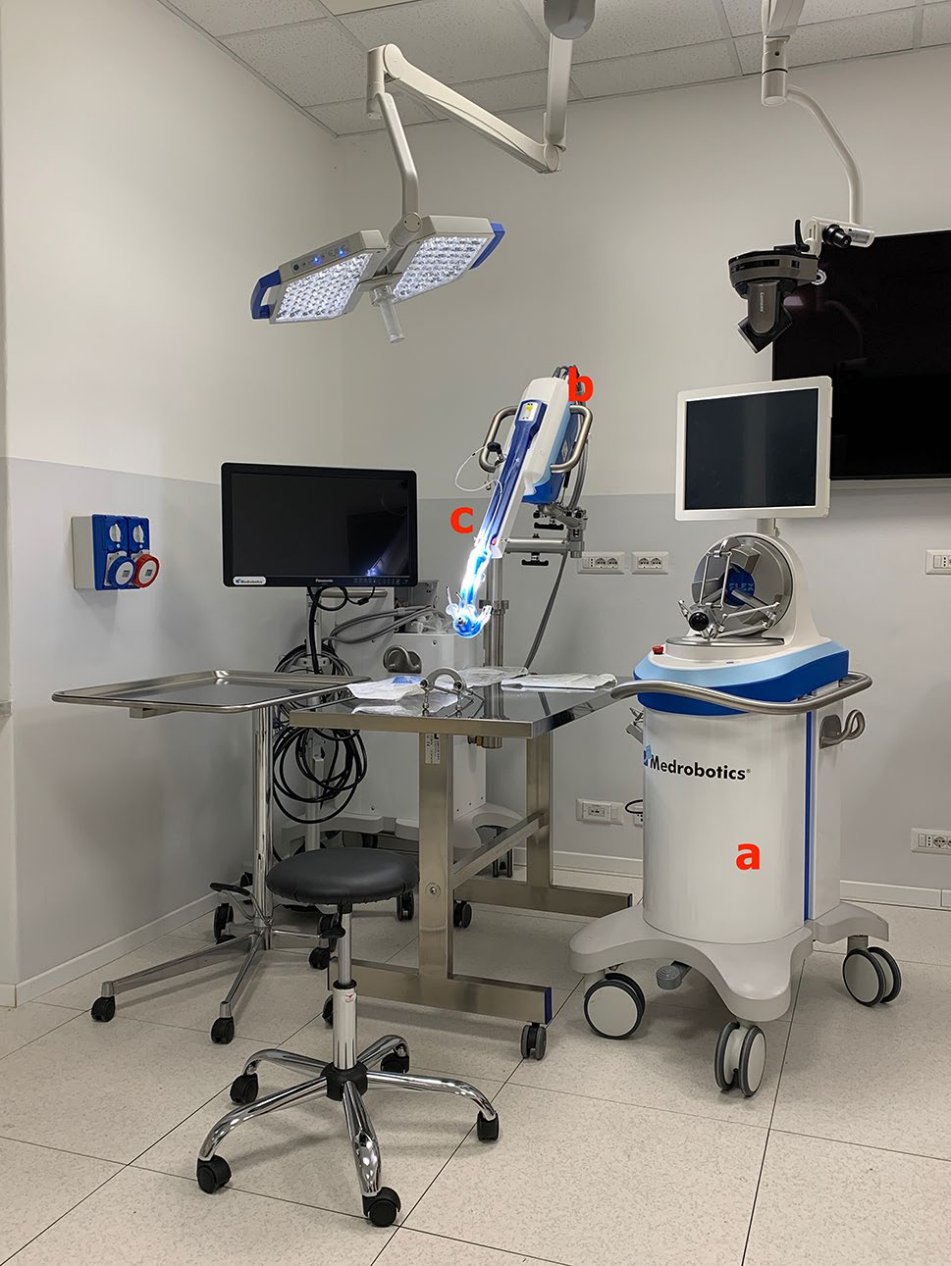
Flex^®^ Robotic System consisting of two transportable main units and a flexible robotic endoscope: **a** Console, and Flex^®^ Drive, **b** Flex^®^ Cart and Base, **c** Flex^®^ Drive

**Fig. 2:**
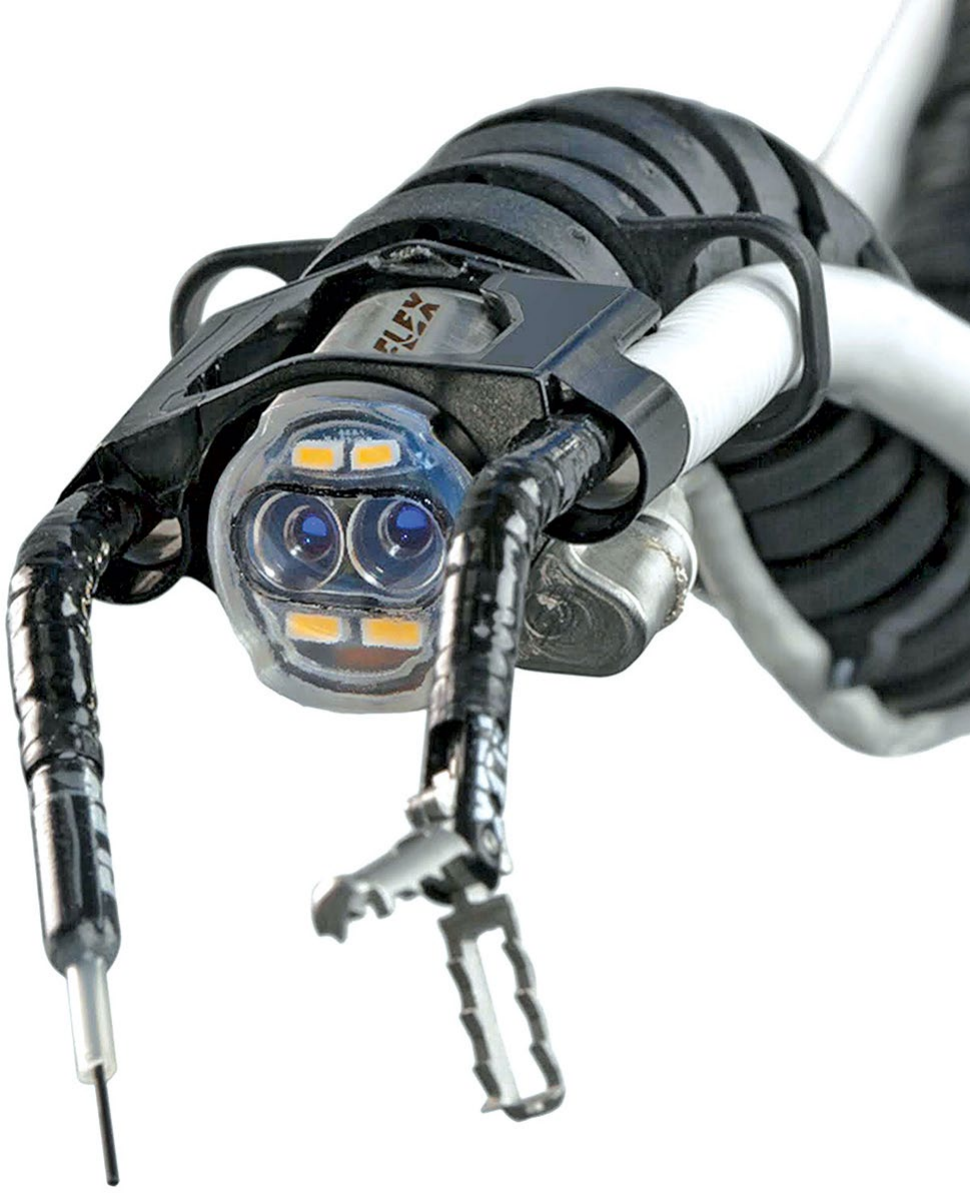
The robot's flexible distal end including two working channels with a needle-knife and grasper

The device is mainly composed of two units and a flexible robotic endoscope (Fig. 1) (Video 1):

- Flex^®^ control console (Fig. 1a). The control unit of the Flex^®^ Robotic System is geared up with a haptic controller, joystick type, which can be moved with precision in space in three dimensions.
- Flex^®^ Cart and Base. The Flex^®^ Cart (Fig. 1b) is the other unit that makes up the Flex^®^ Robotic System that carries the base and provides steadiness to the Flex^®^ Drive (Fig. 1c) to pass instructions by wire via software from the console to the flexible robotic arm.
- Flex^®^ Drive. The working facets of the Flex^®^ Robotic System used in the colorectal surgical procedures are represented (Fig. 2). A miniaturised HD-3D digicam is placed at the tip of the flexible robotic arm. While the digicam is reusable, all the different tools are single use. The two white tubes at the sides of the digicam are working channels placed at 3 and 9 o’clock. These accommodate flexible surgical instruments = 3 in surgical procedures in triangulation with the digicam camera which has a 0° lens.

**Fig. 3:**
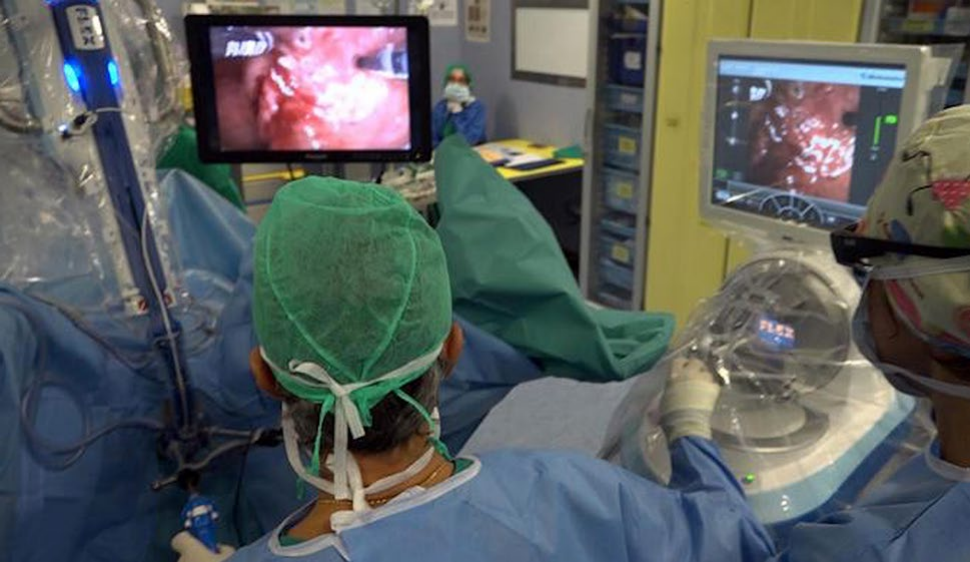
The surgeon, close to the patient, works through 2 dedicated, flexible instruments introduced through the operative channels placed on the sides of the camera, which allow the surgical manipulation of the tissues

During the procedure, the surgeon, next to the patient, works with these two dedicated flexible instruments inserted via the working channels positioned on the two sides of the camera, which allows. surgical manipulation of the tissues while at the same time having close access to the console (Fig. 3).

### Features

The Flex^®^ Robotic System acquired the CE mark for transanal applications (rectum and distal colon) in 2016. The System additionally has United States Food and Drug Administration (FDA) clearance since 2017. In the distal section that houses the camera, the Flex^®^ Drive has a diameter of 28 mm and it is managed by the haptic controller in the console. The console is positioned right next to the operator and at the same time, the surgeon operates specially designed laparoscopic-style pistol-grip instruments. This non-robotic equipment attains the same versatility of a robot, as they can produce working angles away from the device axis, permitting triangulation. In addition, the device incorporates a few bending instruments, 2 mm or 3.5 mm in calibre, such as needle holders, grasping forceps, and monopolar tipped or laser holder coagulation instruments.

The Flex^®^ Robotic System with Flex^®^ Colorectal Drive is anchored transanally. In this configuration, the Flex^®^ Drive is located at 12 o’clock, and a 5–10 mm trocar is placed at 6 o’clock and is linked to a high-flow insufflator for secure pneumatic distension. At 3 and 9 o’clock, metallic conduits are positioned via which flexible instruments are advanced. Finally, the complete machine is fixed to the working desk rail through supports to ensure the steadiness of the platform.

The device is designed for use by a single surgeon. The surgeon first establishes an area of view by positioning the Flex^®^ Drive with the built-in HD camera. Then he/she uses flexible surgical instruments to perform the dissection. The Flex^®^ CR Drive is coupled to a dedicated metallic anoscope anchored to the table (see Video 1), whilst the two working channels are also anchored to the anoscope. Once the device is docked, the operator uses the Flex Control console to navigate inside the lumen and position the Flex Drive closer to the operative target. The Flex^®^ Drive advances with controlled actions of 3 or 5 cm at a time, in absolute safety, until the target's visualisation. Once the operative area is defined, the surgeon uses the two flexible surgical instruments to perform the entire excision, while the optic can be adjusted at any time.

**Fig. 4:**
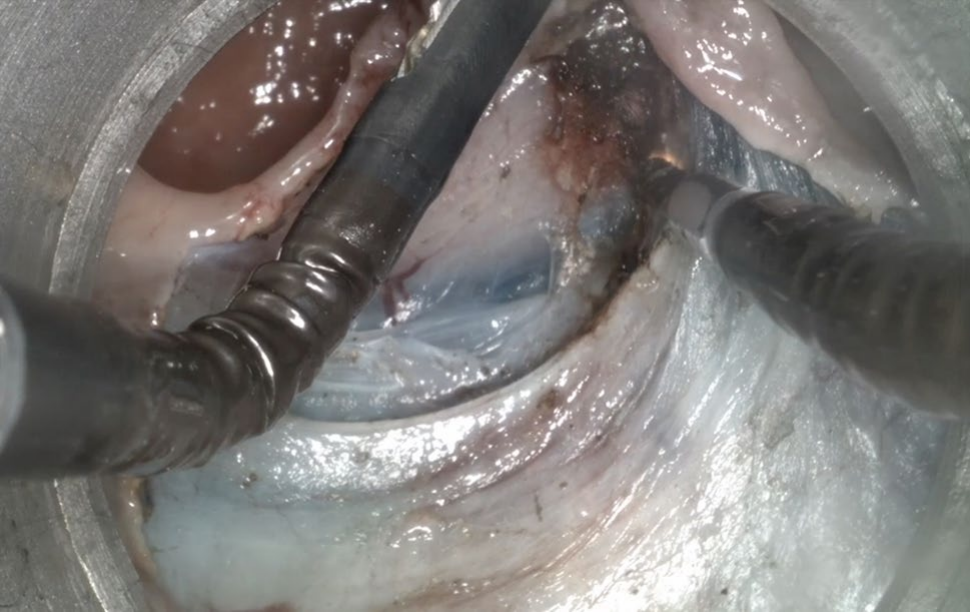
Endoscopic submucosal dissection in the upper rectum anterior wall

In the case of ESD, saline solution with methylene blue is injected into the submucosal layer. Then under counter traction with a grasper, submucosal dissection is performed using a monopolar spatula. A high-frequency generator is used beneath the equal settings used for traditional endoscopic surgery, such as 25 W for coagulation energy. The spatula allows blunt dissection just above the muscularis propria layer, which is entirely spared (Fig. 4). In these cases, suturing is regarded as no longer mandatory.

Depending on the preoperative evaluation of the role and extension of the neoplasm, the patient can be positioned both supine (much more comfortable) for posterior lesions or prone (less comfortable but safer) in case of anterior lesions, mainly if the lesion is located above the peritoneal pouch. This is because if a perforation of the rectal wall occurs, you keep away from small bowel loops shedding interior the rectum, as we realised from the TEM experience.

Since we feel this technique is still part of a learning curve, we opt for general anaesthesia, even though selected indications may be carried out under spinal anaesthesia [14].

The most extended range of the endoscope from the anal margin is presently 17 cm.

## Results

### Technique and technology assessment

We performed the first case with the monopolar high frequency (HF) needle in the right hand and the fenestrated grasper in the left hand. This was a sessile neoplasm of about 25 mm in diameter, at about 7 cm from the anal verge, that underwent a full-thickness excision. Our impression was that the needle was difficult to control, and this created a risk of bowel perforation during dissection. Therefore, we replaced it with a monopolar spatula in all the following cases. Similarly, we felt that the fenestrated grasper was a bit * due to teeth along the jaws. So, we preferred to use the Maryland grasper, an electrified monopolar grasper, in case we needed to control bleeding. After the first 12 cases, a bipolar Maryland became available and was routinely used.

After the first six cases, we changed the design of the rectoscope, which was shaped like a bowler hat, derived from the design of some TAMIS ports. We thought this was unnecessary and could be responsible for damage to the anal sphincter and therefore asked Medrobotics R&D Department to modify the shape to a cylinder.

In all cases, we used an 18 Fr Nelaton tube as a suction tube inserted through the central operating channel. This was inserted through the same central trocar/cannula, which was used for carbon dioxide insufflation.

### Series

Between October 2018 and February 2020, 26 patients with rectal disease underwent transanal local excision with the Flex^®^ Robotic System. Thirteen were males. The median age was 72 years (range 54–89 years). The median body mass index was 26.4 kg/m^2^ (range 20.0–33.3 kg/m^2^).

The indication for surgery was an adenoma in 22 cases (84.6%) and an adenocarcinoma in 4 (15.4%). In two of these four adenocarcinomas cases, full-thickness local excision was indicated after a piecemeal endoscopic resection of unknown cancer at the time of endoscopic excision. The median distal distance from the anal verge was 6 cm (range 2–16 cm). The median proximal distance from the anal verge was 10 cm (range 4–20 cm).

### Intraoperative results

All procedures were performed under general anaesthesia. Sixteen patients (61.5%) were in a supine position, and 10 (38.5%) in a prone position. In 14 cases (53.8%), the procedure consisted of a full-thickness rectal wall excision, and in 12 (46.2%) it consisted of a submucosal dissection. All full-thickness excisions were completed with a double running suture. A first suture started from the midline of the defect towards the patient's right, a second one from the left edge towards the midline of the defect. Nine (75%) of the submucosal dissections were also completed with a running suture. In all cases, we used a V-Lock 3/0 90 15 cm long thread (Medtronic, Minneapolis, MN, USA).

The median operating time was 115 min (range 45–360 min). An unintentional opening of the peritoneum was observed in one case (3.8%) treated with direct suturing. In six patients (21.4%), it was necessary to convert to standard TEO procedure (Karl Storz Endoskope GmbH, Tuttlingen, Germany): in two cases (7.7%), this was necessary to complete the dissection, in four cases (15.4%) to complete suturing the defect.

No procedure required conversion to abdominal surgery. Intraoperative bleeding was always negligible. No intraoperative blood transfusion was needed.

### Postoperative results

There was no 30-day mortality. The 30-day morbidity rate was 11.5% (3/26). None of the patients required further abdominal surgery within 30 days. One patient had a substenosis of the anastomosis after a full-thickness excision, which required endoscopic balloon dilatation. After a submucosal dissection, two patients experienced severe bleeding, both requiring blood transfusion, and in one case even further transanal surgery. There were no cases of urinary retention. The median length of hospital stay was 3 days (range 1–10 days).

### Pathology results and staging

The median surface area removed was 17.5 cm^2^ (range 4–56 cm^2^). Histological examination of the surgical specimens demonstrated an adenoma in 16 cases (61.5%), and a carcinoma in 8 (30.8%). The two remaining patients (7.7%), operated on following incomplete endoscopic polypectomy without histology-proven clear resection margins, showed no residual disease.

Postoperative staging of resected adenocarcinomas was six pTis, one pT1sm1 and one pT1sm2.

Positive resection margins were detected in 15.4% (4/26) patients overall in all cases involving dysplastic areas. No specimen fragmentation occurred.

At a minimum median follow-up of 12 months (range 12–22 months), two recurrences were observed: one was detected in the patient with pT1sm2 adenocarcinoma, the other in the patient with the largest lesion excised, an adenoma with high-grade dysplasia. In addition, one patient with previous piecemeal EMR and no remaining disease after local excision had a recurrent adenocarcinoma 18 months after the index surgery, and was treated with total mesorectal excision. No further recurrences were observed.

## Discussion

For a long time, colorectal neoplasms were considered curable only via abdominal surgery. Forty years ago, TEM opened the way for the use of natural orifices for treatment of rectal lesions. The technique allowed the combination of a less invasive transanal approach with low recurrence rates. This was attributed to the enhanced visualisation of the surgical field, which allowed more precise dissection. The TEM technique showed excellent results for the treatment of large benign sessile neoplasms and early rectal cancers. Nevertheless, it is limited by the rigid structure of the instrumentation that does not allow it to reach above the recto-sigmoid junction.

Recently, there has been terrific progress in new robotics with developing robotic constructions that can engage with the surroundings in an inherently natural way [15–18]. Technological trends include snake-like robots, reconfigurable and self-assembling robots, robotic capsules, self-propelling robots, and smooth and inflatable robots [19–21]. These applied technologies have been developed to attain the goal of reaching a far-off target inside an inaccessible visceral space, such as the colon. All of these have their merits. However, they have shortcomings when considered for use inside a long and narrow colon.

The Flex^®^ Robotic System consists of a robotic steerable endoscope advanced into the bowel, ignoring gravity due to an internal mechanism and carrying a steady surgical platform. Two flexible mechanical arms move parallel to the endoscope. The fundamental indication to use the Flex^®^ Robotic System is the dissection and excision of colorectal neoplasms at an early stage. The system tries to overcome the current limitations of the standard endoscopic techniques EMR and ESD using tissue manipulation and counter traction [22, 23].

The Flex^®^ Robotic System represents the opening of a new frontier of surgery. It is the world's first flexible robotic surgical platform with an orientable and shapable robotic telescope for scar-less surgery. The Flex^®^ Robot System aims to provide surgeons with the unique capacity to operate in hard-to-reach anatomical areas, otherwise inaccessible with straight, inflexible surgical instruments. This should permit a higher number of patients to obtain the advantages of minimally invasive endoluminal surgery.

Parallel to our clinical experience, we actively participated in technology assessment, which led to the modification, development, and implementation of tools according to our clinical experience. This activity included briefing and debriefing meetings with engineers before and after each clinical session, as well as extensive lab activity for testing on ex vivo models, including cadaver labs. Significant achievements reached were (1) a more ergonomic shape of the rectoscope, now easier and less traumatic to introduce through the anus, (2) a bipolar forceps fundamental in case of major bleeding control, (3) a steerable cannula for suction, shaped with the same pistol handle of the other instruments, that can accommodate standard accessories for flexible endoscopy, such as injection needles and HF knives for dissection, and fix their length of insertion, so that you can transform them into steerable independent precision instruments rather than passive ones. This latest option has not yet been tested in clinical cases. Several other improvements are needed and are on the way. First, a fenestrated grasper, also bipolar, for a more gentle and less traumatic tissue handling. Second, a self-adjusting needle holder to improve the dexterity of the manoeuvre of suturing. Third, an easier way to lock the silicon seal of the endoscope to the rigid rectoscope, sometimes tricky and possible cause of air leak. Fourth, U-shaped arms to fix both the rectoscope and the other side of the two instruments to the rails of the operating table; this would make it easier to position the patient prone, avoiding conflicts with the patient's legs.

We are currently experimenting in our lab with a more extended version of the endoscope, now capable of reaching 30 cm, with an increased dexterity, which we expect to pass through the more angulated recto-sigmoid junctions extending this way the application to the sigmoid and descending colon. A major reshaping of the entire system is on the way in the medium term. Not only will the length of the endoscope be increased, the external diameter will be reduced, not only of the endoscope itself but also of the overall size of the robot, moving the two working channels at 10 and 2 o'clock. This will also reduce the risk of conflicts, allowing a better approach to the target tissue and preventing obstruction to the view.

**Fig. 5:**
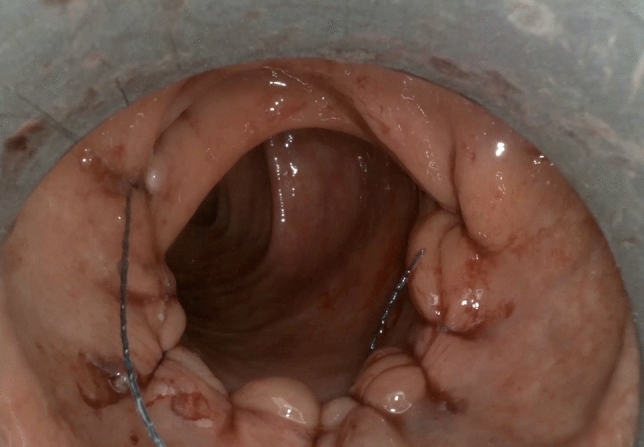
The final image of a running suture with barbed suture after a semi circumferential full-thickness excision

Although the present series represents a work in progress with constant developments of new features, we were able to resect superficial neoplasms up to 8 cm in largest diameter, entailing up to 1/2 of a circumference and extending up to 20 cm from the anal verge, measured with the aid of rigid endoscopy. In fact, this represents the largest series of consecutive transanal procedures performed on humans with this novel technology at a single centre. Our long experience both with the original TEM (Richard Wolf, Knittlingen, Germany), and then with theTEO (Karl Storz, Tuttlingen, Germany) equipment, allowed us to make acritical appraisal of the Flex^®^ Robotic System The precision of HD-3D visualisation and the easy gestures of the committed grasper and spatula, mechanical but flexible, permit the choice of both for an ESD in case a clear plane above the muscle layer is recognisable or for a full-thickness excision if not. Unfortunately, the reliability of preoperative imaging work-up is low [24–26]. Therefore, about 10% of supposed benign lesions turn out to be invasive cancers. Here, after changing the method into a full-thickness excision, the Flex^®^ Robotic System permits reliable full-thickness defect suturing (Fig. 5). An ideal closure of the wound is usually viable in these instances, avoiding the danger of bleeding and lowering the hazard of late perforations. Here too, after a quick learning curve and standardisation of the technique, it is a procedure frequently used to complete an average of about 1 h and a half of inclusive of suturing.

Nevertheless, we are quite disappointed with the clinical results we report. The dexterity of the tools should theoretically allow precision surgery. Instead, we needed to convert about one-fourth of the procedures at a certain point to a standard TEO technique. Furthermore, we registered a 15% rate of R1 resections, with two of these patients requiring further procedures. This is relatively high when compared with our TEM experience [27]. This is surely due in part to the fact that our current series represents a learning phase. Nevertheless, it was our expectation that the increased dexterity would allow immediate better results in en bloc R0 resections and, therefore, a lower recurrence rate. Consequently, we believed but could not confirm what has recently been affirmed by other authors [28], i.e. that the robotic ESD is capable of appreciably decreasing technique time and increasing the complete resection rate for ESD. Nowadays, the gain in comparison to TEM is limited.

Further improvements should not only focus on conceiving longer devices to reach lesions above the recto-sigmoid junction. More important is the necessary progress in precision surgery, with increased dexterity and user-friendliness, to significantly reduce the number of incomplete resections and perioperative complications. Current limitations of the existing platform show that further development in the dexterity of both the endoscope and the flexible instruments, and the availability of more tools is required. Moreover, it will be fundamental to increase the flexibility of the endoscope so as to allow perfect positioning in front of the target lesion, together with better visualization, not necessarily 3D but allowing a wider view of the surgical field. On the other hand, the use of robotic instruments rather than just flexible surgical tools would allow dissection to be more precise In fact, although instruments are designed mainly for the suturing phase, this in our experience was not always possible, whilst becoming fundamental when working inside the peritoneal sac for the risk of perforation through the wall into the peritoneum.

## Conclusions

Our personal experience confirms that although flexible robotic technologies are expected to increase the possibility of the overall performance of difficult dissection inside the gastrointestinal lumen, the current state of the art of technology is still insufficient to overcome the limits of existing rigid platforms. Therefore, further improvements are mandatory to allow surgery primarily based on tissue manipulation, traction, and counter traction.

## Supplementary Information

Below is the link to the electronic supplementary material.

Supplementary file1 (MP4 150406 KB)

## Data Availability

The datasets analyzed during the current study are available from the corresponding author on reasonable request.
